# Injection Laryngoplasty as a Means of Resolving Symptomatic Vocal Cord Immobility After Congenital Heart Surgery

**DOI:** 10.1093/icvts/ivag130

**Published:** 2026-05-05

**Authors:** Mitchell C Haverty, Courtney Long, Alyssia Venna, Manan Desai, Aybala Tongut, Bao N Puente, Yves d’Udekem, Pamela Mudd

**Affiliations:** Division of Cardiovascular Surgery, Children’s National Hospital, Washington, DC, 20010, United States; Division of Hearing and Speech, Children’s National Hospital, Washington, DC, 20010, United States; Division of Cardiovascular Surgery, Children’s National Hospital, Washington, DC, 20010, United States; Division of Cardiovascular Surgery, Children’s National Hospital, Washington, DC, 20010, United States; Division of Cardiovascular Surgery, Children’s National Hospital, Washington, DC, 20010, United States; Division of Cardiac Critical Care, Children’s National Hospital, Washington, DC, 20010, United States; Division of Cardiovascular Surgery, Children’s National Hospital, Washington, DC, 20010, United States; Division of Otolaryngology, Children’s National Hospital, Washington, DC, 20010, United States

**Keywords:** injection laryngoplasty, cardiac surgery, aspiration, dysphagia, vocal cord immobility, vocal cord medialization

## Abstract

**Objectives:**

Vocal cord immobility immediately following cardiac surgery is associated with reduced airway protection, dysphagia and aspiration in infant and paediatric populations. Early vocal medialization shows promise in optimizing feeding outcomes and swallowing mechanics. We aim to demonstrate the outcomes of injection laryngoplasty in patients with symptomatic vocal cord immobility after paediatric cardiac surgery.

**Methods:**

Data from cardiac surgery patients who underwent injection laryngoplasty between January 2021 and December 2024 at a single-centre institution were reviewed. Data was analysed following the implementation of a vocal cord injury protocol, including laryngeal ultrasound screening, preoperative dysphagia assessment, injection laryngoplasty and postoperative dysphagia evaluation as medically appropriate.

**Results:**

Thirty-one patients underwent injection laryngoplasty after diagnosis of symptomatic unilateral vocal cord immobility with objectively identified dysphagia a median of 10 days (2-63) after surgery. A postoperative endoscopic or fluoroscopic swallow study was conducted with dysphagia improving or resolving in 26 of 31 patients post-injection. Four patients were discharged with gastrostomy tubes. Seven patients had a documented complication, including stridor, escalation of care setting, and additional required procedures. These were short-lived and often associated with underlying congenital heart disease and unrelated to vocal cord injection.

**Conclusions:**

Injection laryngoplasty as an intervention for vocal immobility and dysphagia following cardiovascular surgery is a low-risk procedure, allowing safer advancement of oral diet in most patients prior to discharge. We postulate it also improves dysphagia in the subacute phase and may lead to longer-term benefit in glottic protection.

## Graphical abstract

**Figure:**
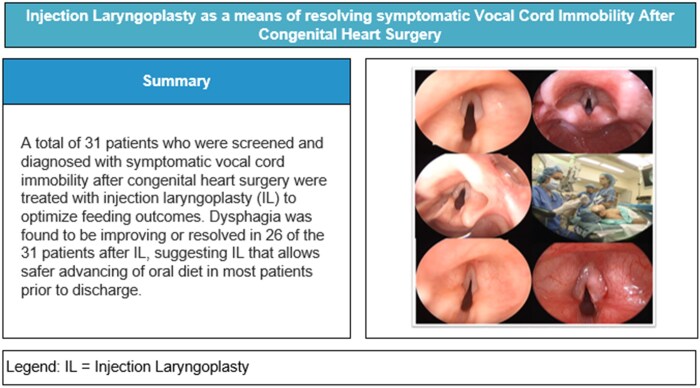

## INTRODUCTION

Vocal cord immobility (VCI), resulting from intraoperative stress to the recurrent laryngeal nerve (RLN) has a wide variability of incidence, with research yielding ranges from 8% to 59% after paediatric cardiac surgery and rates as high as 77% for neonatal surgeries specifically involving the aortic arch.^1^^,^^2^ Unilateral or bilateral VCI due to paralysis or paresis has been found to result in increased postoperative morbidity, including pharyngeal dysphagia with associated tracheal aspiration, increased lengths of stay and higher incidence of gastrostomy tube placement.^3^^,^^4^ The rate and time to recovery from VCI following paediatric cardiac surgery are variable. Approximately 68% of infants with VCI after paediatric cardiac surgery can recover in an average of 3 months, but other reviews report that recovery can require over twelve months.^5–7^

Injection laryngoplasty (IL) is an injection of dissolvable filler such as hyaluronic acid or carboxymethylcellulose gel into the paralaryngeal space to medialize the affected vocal cord, which is increasingly utilized as a treatment option for VCI and airway invasion in paediatric populations.^8^^,^^9^ Data for paediatric IL after diagnosis of VCI have suggested that the procedure is safe and beneficial, assisting with aspiration resolution and vocal strength as well as a greater than 80% improvement in swallow function.^10^^,^^11^ To address such significant implications, a pilot programme at Children’s National Hospital was established between the Heart Center and the Divisions of Otolaryngology and Hearing and Speech. This pilot programme was developed to optimize safe feeding outcomes in cardiac surgery in the setting of unilateral VCI.

Study Aim: Assess whether postoperative swallowing and feeding outcomes improved in the setting of vocal cord immobility following injection laryngoplasty in a paediatric cardiovascular surgery cohort.

Hypothesis: Airway invasion will resolve or improve, and oral intake will increase after IL.

## METHODS

This is a single-centre Children’s National Institutional Review Board approved (IRB# Pro00015566) retrospective review of data of children who received IL during the inpatient stay following cardiac surgery between January 2021 and December 2024. A waiver of consent for the acquisition of data was granted from the IRB due to the minimal risk nature of the study. Data storage from research participants is consistent with requirements outlined in the WMA Declaration of Taipei. An ethics committee approved the establishment and monitoring of this database. CPT code searches and locations of service were used to identify patients who were kept in an ongoing database. Injection laryngoplasty was completed under general anaesthesia with suspension laryngoscopy utilizing either hyaluronic acid or carboxymethylcellulose gel depending on otolaryngologist preference during a stay for cardiac surgery. Otolaryngology follow-up was conducted during an outpatient clinic, where vocal cord mobility was assessed using flexible laryngoscope. Data collected included sex, age at time of cardiac surgery, gestational age, presence of genetic abnormalities (either a chromosomal abnormality or syndrome as per STS definition), cardiac physiology and operative diagnosis, preoperative disposition, and specified injection post-IL complications, postoperative disposition, swallowing assessments, and feeding outcomes.

### Definitions

Airway invasion was defined as deep laryngeal penetration of material just above/or to the level of the vocal folds, while tracheal aspiration was defined as material entering the subglottic trachea. This was diagnosed on Flexible Endoscopic Evaluation of Swallowing (FEES) in the anterior-posterior view or laterally on a Modified Barium Swallow Study (MBSS). Patients diagnosed with bilateral VCI had resolution to single-sided VCI prior to IL. Improvement in dysphagia was assessed by the Penetration-Aspiration Scale (PAS)^12^ for airway invasion and the Functional Oral Intake Scale (FOIS)^13^ adapted for infants for oral volumes. Per Os (PO) feeding was defined as nutrition taken by mouth. Early outcomes were defined as the postoperative inpatient course leading up to discharge. Late outcomes were defined as the first otolaryngology follow-up after disposition, occurring within 8-12 weeks after discharge.

### Statistical analysis

Descriptive analysis was performed on the cohort. Continuous variables were analysed using the median and interquartile ranges (IQR). Categorical variables were analysed using frequency and percentage. Categorical variables were assessed by Fisher’s exact test, while continuous variables were assessed by Mann–Whitney *U* tests. All assumptions were met; if not, appropriate transformations were performed. All statistical analyses were performed using R software (version 4.4.3). Exposure groups were patients with isolated aortic arch interventions and patients with genetic abnormalities. The primary outcome assessed was feeding status at the time of discharge.

## RESULTS

Patient characteristics, discharge feeding plans and early-to-late outcomes are included in **Tables 1 and 2**. A total of 140 patients were assessed for vocal mobility following cardiac surgery by either ultrasound or bedside endoscopy. Out of 140 patients who were screened, 70 (50.0%) were found to have vocal cord injuries. A total of 33 (23.6%) received vocal cord injections, of which 31 (22.1%) underwent IL before discharge from the hospital; the other 2 patients were excluded because IL was performed after initial admission. Patient screening is summarized in **Figure S1**. The median age (IQR) at IL was 38.0 (28-72) days. Cardiac surgery included 22 (71.0%) aortic arch repairs (8 of which were isolated), 5 (16.1%) patients with single ventricle palliations, 2 (6.5%) ventricular septal defects repairs, 1 (3.2%) vascular ring and 1 (3.2%) ECMO cannulation. Genetic abnormalities were present in 7 (22.6%) patients.

**Table 1.** Demographic and Clinical Data of Patients With Genetic Abnormalities Compared to Patients Without Genetic Abnormalities

	Total	Genetic abnormalities	No genetic abnormalities	*P*-value
**Number of patients, *n* (%)**	31 (100%)	7 (22.6%)	24 (77.4%)	
**Female sex, *n* (%)**	13 (56.5%)	3 (42.9%)	10 (41.7%)	1.000
**Gestational age (weeks), median (IQR)**	38 (37-40)	37 (36-38)	39 (38-40)	.038
**Age at cardiac surgery (days), median (IQR)**	19 (7-42.5)	30 (10-35)	18.5 (7-50)	.849
**Surgical repair**				.324
Aortic arch repair	22 (71.0%)	4 (57.1%)	18 (75.0%)	
Single ventricle	5 (16.1%)	3 (42.9%)	2 (8.3%)	
Ventricular septal defect	2 (6.5%)	0	2 (8.3%)	
Vascular ring	1 (3.2%)	0	1 (4.7%)	
VA ECMO	1 (3.2%)	0	1 (4.7%)	
**Vocal cord injury diagnosis, *n* (%)**				.212
Left	27 (87.1%)	5 (71.4%)	22 (91.7%)	
Right	2 (6.5%)	1 (14.3%)	1 (4.2%)	
Bilateral (with single sided resolution)	2 (6.5%)	1 (14.3%)	1 (4.2%)	
**Time from cardiac surgery to diagnosis (days)**	10 (6-10)	11 (8-22)	9.5 (5-15)	.267
**Age at injection laryngoplasty, median (IQR)**	38 (28-72)	48 (29-85)	37 (27-67)	.810
**PreOp aspiration, *n* (%)**				.489
Yes	23 (88.5%)	4 (80.0%)	19 (90.5%)	
No	3 (11.5%)	1 (20.0%)	2 (9.5%)	
Not assessed	5	2	3	
**Time from diagnosis to injection (days), median (IQR)**	7 (5-12)	12 (7-14)	6 (4-10)	.103
**Injection material**				1.000
Carboxymethylcellulose-Prolaryn gel	23 (74.2%)	5 (71.4%)	18 (75.0%)	
Hyaluronic acid-Juvaderm	8 (25.8%)	2 (28.6%)	6 (25.0%)	
**Injection volume (mL), median (IQR)**	0.15 (0.10-0.20)	0.15 (0.13-0.20)	0.15 (0.10-0.20)	.984
**Additional airway conditions, *n* (%)**				1.000
Yes	9 (29.0%)	2 (28.6%)	7 (29.2%)	
No	22 (71.0%)	5 (71.4%)	17 (70.8%)	

**Table 2.** Demographic and Clinical Data of Patients With Isolated Arch Repair Compared to Patients With Other Surgical Repairs

	Total	Isolated arch	Other repairs	*P*-value
**Number of patients, *n* (%)**	31 (100%)	8 (25.8%)	23 (74.2%)	
**Female sex, *n* (%)**	13 (56.5%)	3 (37.5%)	10 (43.5%)	1.000
**Gestational age (weeks), median (IQR)**	38 (37-40)	39 (38-40)	38 (37-40)	.368
**Age at cardiac surgery (days), median (IQR)**	19 (7-42.5)	18 (7-35)	19 (7-47)	.682
**Genetic abnormalities, *n* (%)**	7 (22.6%)	0	7 (30.4%)	.146
**Vocal cord injury diagnosis, *n* (%)**				.719
Left	27 (87.1%)	7 (87.5%)	20 (87.0%)	
Right	2 (6.5%)	0	2 (8.7%)	
Bilateral (with single sided resolution)	2 (6.5%)	1 (12.5%)	1 (4.3%)	
**Time from cardiac surgery to diagnosis (days)**	10 (6-10)	6 (5-9)	11 (6-19)	.126
**Age at injection laryngoplasty, median (IQR)**	38 (27.5-72)	33 (21-66)	46 (29-85)	.308
**PreOp aspiration, *n* (%)**				1.000
Yes	23 (88.5%)	6 (85.7%)	17 (89.5%)	
No	3 (11.5%)	1 (14.3%)	2 (10.5%)	
Not assessed	5	1	4	
**Time from diagnosis to injection (days), median (IQR)**	7 (5-12)	7 (5-12)	7 (5-12)	.912
**Injection material**				.642
Carboxymethylcellulose-Prolaryn gel	23 (74.2%)	7 (87.5%)	16 (69.6%)	
Hyaluronic acid-Juvaderm	8 (25.8%)	1 (12.5%)	7 (30.4%)	
**Injection volume (mL), median (IQR)**	0.15 (0.10-0.25)	0.13 (0.10-0.18)	0.15 (0.10-0.20)	.803
**Additional airway conditions, *n* (%)**				.660
Yes	9 (29.0%)	3 (37.5%)	6 (26.1%)	
No	22 (71.0%)	5 (62.5%)	17 (73.9%)	

Twenty-nine (93.5%) patients were screened with both ultrasound and bedside endoscopy; 1 (3.2%) underwent only ultrasound, and 1 (3.2%) underwent only endoscopy. A total of 21 ultrasounds and endoscope diagnostics agreed, while 8 yielded different results. In these 8 cases, 7 were incorrectly identified by ultrasound and 1 case was incorrectly identified by endoscopy.

A pre-IL Fibreoptic Endoscopic Evaluation of Swallowing (FEES) or Modified Barium Swallow Study (MBSS) was conducted in 26 (83.9%) of the 31 total patients, most of which yielded airway invasion resistant to standard age-appropriate manoeuvres. Those that did not receive a swallowing assessment were deemed inappropriate for oral intake at the time of clinical evaluation, though they had identified risk factors such as wet cough, persisting chest congestion and/or reflux symptoms where aspiration of stomach contents or secretions warranted concern in the setting of VCI. Post-IL swallowing assessments were conducted in 26 patients (83.9%).

Post-IL complications were experienced by 7 (22.6%) patients, including increased work of breathing (*n* = 2), intermittent or persistent stridor (5), heart failure (1) and sepsis (1). All complications were brief and self-resolving. Patients were discharged a median of 28 days (22-48) after cardiac surgery and 11 days (7-18) after IL (**Tables 3 and 4**). Patients with genetic abnormalities had a median discharge time of 19 (14-39) days compared to those without at 8 (6-13) days after IL (*P* = 0.007). Patients who had isolated aortic arch repair had a median time to discharge of 8 days (6-12) compared to a median time of 12 (7-20) days for those with other repairs (*P* = 0.131). Dysphagia improvement was demonstrated in 26 (83.9%) patients from original PAS and/or FOIS scores. Of the 26 demonstrating improvements, 19 (73.1%) exhibited increases in both PAS and FOIS scores. Two (2.7%) patients had improvements in PAS score only, while 5 (19.2%) patients had improvements in FOIS scores only. Of the 5 patients with FOIS score improvements, 3 presented with normal PAS postoperative scores, although they could not be counted as technical increases given a preoperative swallow study was not done for comparison (**Table S2**). A total of 19 (61.3%) patients were discharged with full PO intake with specified feeding modalities for safe practice after IL, while 8 (25.8%) patients were discharged home with balanced oral and gavage feedings. Four (12.9%) patients received gastrostomy tubes due to feeding intolerance (3), failure to thrive (2), or gastroesophageal reflux (1). Two (28.6%) of the patients with genetic abnormalities and 2 (8.3%) without genetic abnormalities were discharged with gastrostomy tubes. No patients with isolated arch repairs were discharged with a gastrostomy tube vs 4 (17.4%) patients with other repairs.

**Table 3.** Early Outcomes After Injection Laryngoplasty of Patients With Genetic Abnormalities Compared to Those Without Genetic Abnormalities

	Total	Genetic abnormalities	No genetic abnormalities	*P*-value
**PostOp aspiration, *n* (%)**				1.000
Yes	11 (42.3%)	2 (40.0%)	9 (42.9%)	
No	15 (57.7%)	3 (60.0%)	12 (57.1%)	
Missing	5	2	3	
**Complications, *n* (%)**				1.000
Yes	7 (22.6%)	1 (14.3%)	6 (25.0%)	
No	24 (77.4%)	6 (85.7%)	18 (75.0%)	
**More severe disposition post-injection, *n* (%)**				.407
Yes	2 (6.5%)	1 (14.3%)	1 (4.2%)	
No	29 (93.5%)	6 (85.7%)	23 (95.8%)	
**Discharge feeding, *n* (%)**				.148
PO	19 (61.3%)	5 (71.4%)	14 (58.3%)	
NG-tube	8 (25.8%)	0	8 (33.3%)	
G-tube	4 (12.9%)	2 (28.6%)	2 (8.3%)	
**Time from injection to discharge (days), median (IQR)**	11 (7-18)	19 (14-39)	8 (6-13)	.007
**Time from cardiac surgery to discharge (days), median (IQR)**	28 (22-48)	49 (35-83)	26 (22-40)	.009
**Dysphagia improvement, *n* (%)**	26 (83.9%)	5 (71.4%)	21 (91.3%)	.312

**Table 4.** Early Outcomes After Injection Laryngoplasty of Patients With Isolated Arch Repair Compared to Patients With Other Surgical Repairs

	Total	Isolated arch	Other repairs	*P*-value
**PostOp aspiration, *n* (%)**				.658
Yes	11 (42.3%)	2 (28.6%)	9 (47.4%)	
No	15 (57.7%)	5 (71.4%)	10 (52.6%)	
Missing	5	1	4	
**Complications, *n* (%)**				1.000
Yes	7 (22.6%)	2 (25.0%)	5 (21.7%)	
No	24 (77.4%)	6 (75.0%)	18 (78.3%)	
**More severe disposition post-injection, *n* (%)**				.456
Yes	2 (6.5%)	1 (12.5%)	1 (4.3%)	
No	29 (93.5%)	7 (87.5%)	22 (95.7%)	
**Discharge feeding, *n* (%)**				.519
PO	19 (61.3%)	5 (62.5%)	14 (60.9%)	
NG-tube	8 (25.8%)	3 (37.5%)	5 (21.7%)	
G tube	4 (12.9%)	0	4 (17.4%)	
**Time from injection to discharge (days), median (IQR)**	11 (7-18)	8 (6-12)	12 (7-20)	.131
**Time from cardiac surgery to discharge (days), median (IQR)**	28 (22-48)	22 (8-32)	30 (25-51)	.075
**Dysphagia improvement, *n* (%)**	26 (83.9%)	8 (100%)	18 (78.3%)	.291

Following discharge, 26 (83.9%) patients had otolaryngology follow-up within 2 months. In the 8 patients who were discharged from their admission on nasogastric (NG) tube feeds, 6 (75.0%) had otolaryngology follow-up, while the 2 (50.0%) of the 4 patients discharged on gastrostomy (G) tube feeding received follow-up. A total of 15 patients (48.4%) had full vocal cord recovery after a median time of 119 (4-159) days. Genetic comorbidity did not portend a lower risk of neurological return, as 3 (75.0%) patients with genetic abnormalities recovered fully vs 12 (54.5%) without. Full vocal cord recovery was noted in 5 (71.4%) patients with isolated arch repair, compared to 10 (52.6%) of the cohort who had other repairs. In the 11 patients that did not have full recovery of the vocal cords, 9 (81.8%) patients were on fully PO diets at their last otolaryngology follow-up, 1 (11.1%) patient was feeding with an NG tube, and 1 (11.1%) was partially feeding with a G-tube. Of patients discharged with NG feeding who received follow-up, 4 (66.7%) advanced to full oral feeds, while 2 (33.3%) remained on enteral feeds. Of the remaining patients discharged with gastrostomy feeds who had follow-up, both remained on long-term G-tube feeds. There were no significant differences in follow-up feeding status based on either the presence of genetic abnormalities or aortic arch repair. The median time to full oral intake status post-IL was 8 (5-16) days; patients with genetic abnormalities were 11 (7-15) days, while those without had a median time of 8 (5-25) days. Those with isolated aortic arch intervention had a median of 7 (5-70) days to achieve full oral intake compared to 8 (7-15) days in those with other repairs. No patient required repeat IL for the management of dysphagia, dysphonia, or impaired airway clearance during the follow-up period. Late outcomes are described in **Tables 5 and 6**.

**Table 5.** Late Outcomes After Injection Laryngoplasty of Patients With Genetic Abnormalities Compared to Patients Without Genetic Abnormalities

	Total	Genetic abnormalities	No genetic abnormalities	*P*-value
**Otolaryngology follow-up, *n* (%)**				.062
Yes	26 (83.9%)	4 (57.1%)	22 (91.7%)	
No	5 (16.1%)	3 (42.9%)	2 (8.3%)	
**Full recovery from injury, *n* (%)**				.614
Yes	15 (57.7%)	3 (75.0%)	12 (54.5%)	
No	11 (42.3%)	1 (25.0%)	10 (45.5%)	
Missing	5	3	2	
**Follow-up feeding for non-full recovery, *n* (%)**				1.000
PO	9 (81.8%)	1 (100%)	8 (80.0%)	
NG-tube	1 (9.1%)	0	1 (10.0%)	
G-tube	1 (9.%)	0	1 (10.0%)	
**Follow-up feeding for NG feeders, *n* (%)**				1.000
PO	4 (66.7%)	0	4 (66.7%)	
NG-tube	2 (33.3%)	0	2 (33.3%)	
Missing	2	0	2	
**Follow-up feeding for G-tube feeders, *n* (%)**				1.000
PO	0	0	0	
G-tube	2 (100%)	0	2 (100%)	
Missing	2	2	0	
**Time from discharge to full recovery (days), median (IQR)**	119 (43-159)	16 (11-93)	120 (66-171)	<.001
**Time from injection to full recovery (days), median (IQR)**	131 (55-173)	28 (24-106)	139 (73-184)	<.001
**Full PO diet achieved, *n* (%)**				1.000
Yes	22 (71.0%)	5 (71.4%)	17 (70.8%)	
No	9 (29.0%)	2 (28.6%)	7 (29.2%)	
**Time from injection to full PO diet (days), median (IQR)**	8 (5-16)	11 (7-15)	8 (5-25)	.596
**Time from discharge to follow-up (months), median (IQR)**	2 (0.9-4.2)	0.8 (0.5-2.2)	2.7 (1.0-4.2)	<.001

**Table 6.** Late Outcomes After Injection Laryngoplasty of Patients With Isolated Arch Repair Compared to Patients With Other Surgical Repairs

	Total	Isolated arch	Other repairs	*P*-value
**Otolaryngology follow-up, *n* (%)**				1.000
Yes	26 (83.9%)	7 (87.5%)	19 (82.6%)	
No	5 (16.1%)	1 (12.5%)	4 (17.4%)	
**Full recovery from injury, *n* (%)**				.662
Yes	15 (57.7%)	5 (71.4%)	10 (52.6%)	
No	11 (42.3%)	2 (26.6%)	9 (47.4%)	
Missing	5	1	4	
**Follow-up feeding for non-full recovery, *n* (%)**				1.000
PO	9 (81.8%)	2 (100%)	7 (77.8%)	
NG-tube	1 (9.1%)	0	1 (11.1%)	
G-tube	1 (9.%)	0	1 (11.1%)	
**Follow-up feeding for NG feeders, *n* (%)**				
PO	4 (66.7%)	2 (100%)	2 (50.0%)	.467
NG-tube	2 (33.3%)	0	2 (50.0%)	
Missing	2	1	1	
**Follow-up feeding for G-tube feeders, *n* (%)**				
PO	0	0	0	1.000
G-Tube	2 (100%)	0	2 (100%)	
Missing	2	0	2	
**Time from discharge to full recovery (days), median (IQR)**	119 (43-159)	148 (72-241)	115 (40-121)	.503
**Time from injection to full recovery (days), median (IQR)**	131 (55-173)	162 (76-248)	125 (50-153)	.424
**Full PO diet achieved, *n* (%)**				1.000
Yes	22 (71.0%)	6 (75.0%)	16 (69.6%)	
No	9 (29.0%)	2 (25.0%)	7 (30.4%)	
**Time from injection to full PO diet (days), median (IQR)**	8 (5-16)	7 (5-70)	8 (7-15)	.826
**Time from discharge to follow-up (months), median (IQR)**	2 (0.9-4.2)	4.3 (1.7-6.5)	1.3 (0.9-4.0)	.327

One patient died 135 days following IL due to underlying terminal heart failure.

## DISCUSSION

Our outcomes suggest IL is a successful intervention for VCI after paediatric cardiac surgery, with promising results in mitigating morbidity in swallowing, optimizing anomalies in airway protection and hopefully preventing pulmonary consequences. Our results indicate that 26 of the 31 patients receiving IL demonstrated objective improvements in either PAS or FOIS scores.

Paediatric cardiovascular intervention, particularly near the aortic arch, is a known risk factor of VCI due to the potential stress or injury to the RLN.^4^^,^^14^^,^^15^ Common VCI symptoms include hoarse voice and stridor, though these may often be thought of as related to deconditioning or history of intubation. Dysphagia after cardiac surgery is common and likely related to many factors, including vagal tone changes, deconditioning, and haemodynamic changes.^16^ VCI may lead to dysphagia given the reduced ability to achieve glottic closure to protect the airway and achieve a functional, pressurized swallow.^8^ There are known changes in mechanical swallow function secondary to a diagnosis of VCI, including impaired pharyngeal constriction, glottic incompetence and decreased laryngeal sensation. Such changes directly impact airway closure, tracheal protection, and effective bolus clearance during paediatric deglutition.^10^^,^^17–19^ While not all patients will aspirate a bolus secondary to vocal cord dysfunction, up to 60% may silently aspirate.^20^ Flow rate modulation, side-lying positioning with the affected cord superior and thickened fluid practices can decrease risk during PO.^5^^,^^21^ However, given alterations in swallowing physiology, feeding strategies alone may not be enough to eliminate airway invasion.

Infants with VCI demonstrating deep laryngeal penetration or tracheal aspiration on either a FEES or an MBSS can be at risk for chronic pulmonary aspiration (CPA). Freitag *et al.*^22^ described that CPA can be associated with microbial shifts in the lungs, potentially contributing to development of aspiration associated pathogenesis while Duncan *et al.*^23^ discovered that neonates aspirating breastmilk had higher rates of pulmonary hospitalizations and inflammatory markers. These findings emphasize that aspiration events may translate into respiratory consequences.

It is important to note that a comparative study between patients receiving IL and those without was not conducted, though future research should explore this. Additionally, approximately 20% of patients with genetic abnormalities were shown to have a significantly longer time to discharge compared to those without. Prior literature has demonstrated that individuals with genetic abnormalities have longer lengths of stay following cardiac surgery.^24^^,^^25^ Patients with genetic anomalies had higher rates of airway conditions and a higher proportion of single ventricle disease, both associated with increased postoperative length of stay.^26^^,^^27^ These findings warrant more detailed investigation in future studies.

This research highlights the efficacy of IL to overcome some degree of multifactorial dysphagia. By injecting filler material in the vocal cord, increased bulking of the impaired cord is achieved, allowing the mobile vocal cord to better compensate for glottic closure, reducing or preventing material from entering the airway.^8^ This level of compensation can indirectly assist with the development of more functional feeding patterns, potentially preventing long-term maladaptive feeding experiences in the neonatal population.^28^^,^^29^ We have been so encouraged by early results that we now envision performing this minor surgery to all medically appropriate neonates with identified vocal cord palsy after paediatric cardiac procedures.

## Supplementary Material

ivag130_Supplementary_Data

## Data Availability

The data underlying this article cannot be shared publicly due to the privacy of the individuals involved in the study. The data will be shared on reasonable request to the corresponding author.
