# Supplementary material for: Injection Laryngoplasty as a Means of Resolving Symptomatic Vocal Cord Immobility After Congenital Heart Surgery
Source: Interdiscip Cardiovasc Thorac Surg. 2026 May 5;41(5):ivag130. doi: 10.1093/icvts/ivag130 (PMC13215875; doi:10.1093/icvts/ivag130)
Supplement: ivag130_Supplementary_Data [file ivag130_supplementary_data.docx]

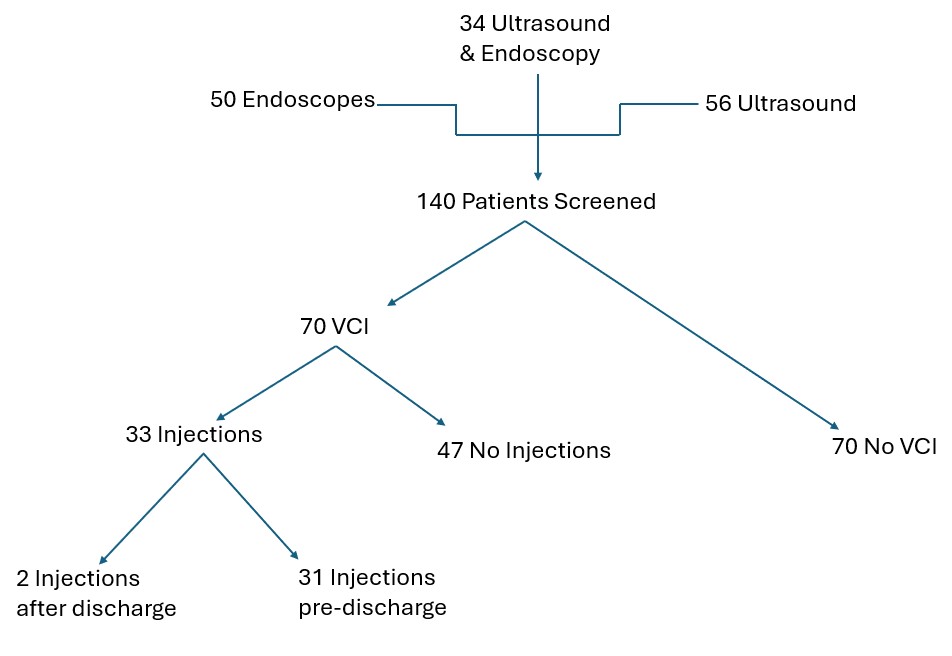


**Supplemental Figure S1**: Flow diagram of the patient selection process, showing how many were screened during the study period and how many were found to have vocal cord immobility.

Abbreviations: VCI: Vocal Cord Injury

| **Patient** | **Genetic Abnormality** | **Isolated Arch** | **Preoperative PAS** | **Postoperative PAS** | **Preoperative**  **FOIS** | **Postoperative FOIS** |
| --- | --- | --- | --- | --- | --- | --- |
| P1 | 0 | 0 | 8 | 1-cup, 8-straw | 4 | 5 |
| P2 | 0 | 0 | 8 | 1-change in flow rate, 8-without | 2 | 4 |
| P3 | 0 | 0 | NA | NA | 2 | 2 |
| P4 | 0 | 1 | clinical bedside aspiration | 1 | 2 | 3 |
| P5 | 0 | 1 | 5 | 2 | 2 | 3 |
| P6 | 0 | 1 | 8 | 1 | 2 | 3 |
| P7 | 0 | 1 | 8 | 1 | 3 | 3 |
| P8 | 0 | 1 | 8 | 8 | 2 | 3 |
| P9 | 1 | 0 | NA | NA | 1 | 1 |
| P10 | 0 | 0 | 8 | 1 | 2 | 3 |
| P11 | 0 | 0 | 8 | 8 | 2 | 2 |
| P12 | 0 | 0 | 8 | 1-flow rate change, 8-fast flow rate | 2 | 3 |
| P13 | 0 | 0 | 8 | 1 | 2 | 3 |
| P14 | 0 | 0 | clinical bedside aspiration | 1 | 2 | 3 |
| P15 | 0 | 0 | 8 | 4 | 2 | 3 |
| P16 | 0 | 0 | 8 | 1 | 2 | 3 |
| P17 | 1 | 0 | 8 | 1 | 2 | 3 |
| P18 | 1 | 0 | 8 | 1-pacing, 8-no pace | 2 | 3 |
| P19 | 0 | 1 | 1 | NA | 2 | 3 |
| P20 | 1 | 0 | 8 | 1-with flow rate change, 8- faster flow | 2 | 3 |
| P21 | 0 | 0 | NA | NA | 2 | 2 |
| P22 | 0 | 0 | 8 | 7 | 2 | 3 |
| P23 | 0 | 0 | 8 | 8 | 1 | 3 |
| P24 | 0 | 0 | 8 | NA | 2 | 1 |
| P25 | 0 | 0 | 8 | 1 | 2 | 3 |
| P26 | 0 | 1 | 8 | 1 | 2 | 3 |
| P27 | 1 | 0 | 8 | 1 | 2 | 3 |
| P28 | 0 | 0 | 8 | 1 | 2 | 3 |
| P29 | 1 | 1 | 8 | 2-with flow rate change, 8-faster flow &thickener | 2 | 3 |
| P30 | 0 | 0 | 8 | 4-with bottle pacing, 8-without | 3 | 3 |
| P31 | 1 | 0 | 5 | 1 | 2 | 3 |

Supplemental Table S2: PAS and FOIS scores of each patient before vocal cord injection and after vocal cord injection. Some patients had split PAS scores depending on the medium of oral intake and have two scores indicated here. For patients that did not have a preoperative swallowing assessment (either a Modified Barium Swallow Study or Flexible Endoscopic Evaluation of Swallowing), preoperative PAS scores could not be assessed.

Abbreviations: PAS = Penetration/Aspiration Scale, FOIS = Functional Oral Intake Scores
